# Editorial: Endoplasmic Reticulum and Its Role in Tumor Immunity

**DOI:** 10.3389/fonc.2015.00252

**Published:** 2015-11-17

**Authors:** Paul Eggleton, Marek Michalak, Edwin Bremer

**Affiliations:** ^1^University of Exeter Medical School, Exeter, UK; ^2^Department of Biochemistry, University of Alberta, Edmonton, AB, Canada; ^3^Laboratory for Translational Surgical Oncology, Department of Surgery, University Medical Center Groningen, University of Groningen, Groningen, Netherlands

**Keywords:** aminopeptidases, autoimmunity, angiogenesis, cancer, genome damage, oxidoreductases, phage display, vaccines

Cancer cells express surface proteins and display antigens that differ from the “norm.” These differences can be exploited to promote a therapeutic antitumor immune response. Specifically, components within the endoplasmic reticulum (ER) play a critical role in deciding which antigenic peptides are presented on the cancer cell surface to immune cells. Furthermore, under stress conditions, certain ER-resident proteins can exit the ER and translocate to the surface. The translocation of such ER proteins to the outside of the cell (1, 2) can lead to modulation of immune responses in cancer (3, 4), autoimmunity (5) and other diseases (6, 7). Normally, proteins undergo a number of ER stress checks for correct folding and, e.g., ability to resist inappropriate oxidation and reduction before secretion. Failing these quality controls leads to ER stress and triggers a series of unfolded protein responses (UPRs) to restore order. In cancer cells, these pathways can be dysregulated opening up the possibility of developing potential therapeutics to target cancer cells (8). In this topic, these various aspects of the ER in tumor immunity are explored in a series of focused review and research articles.

## Behind the “Ion” Curtain

Within the Ca^2+^-ion rich confines of the ER, chaperones, oxidoreductases, aminopeptidases (ERAPs) work industriously for the benefit of the cellular state, regulating signaling to the “outside world.” The calcium channels linking the ER lumen and cytosol act as ER stress gates and chaperones, such as GRP78, act as gate keepers deciding the fate of the cell by their ability to control Ca^2+^ release (9). Alterations in Ca^2+^ homeostasis in the ER can provoke cell stress and trigger one or more UPR coping mechanism pathways, which normally leads to either recovery of a stressed cell or non-inflammatory cell death. However, solid tumors typically thrive in a low oxygen and nutrient environment that usually triggers ER stress. Dicks and coworkers describe corrective UPR strategies that aid malignant cells to survive in this environment, with a focus on GRP78 (10). In brief, GRP78 transcription triggered by ER stress facilitates chromatin remodeling and DNA damage repair and in certain types of malignancies aids survival.

One of the best known immune-regulatory functions occurring within the ER is the assembly of the major histocompatibility complex (MHC)-I/antigen peptide complex. Stratikos and colleagues report on the role of the ER aminopeptidases (ERAP1 and 2) in generating mature antigenic epitopes for loading onto the MHC class I molecules, prior to their transport to the cell surface (11). The authors suggest that both ERAP 1/2 are required for natural killer and T cell-mediated immunity against tumors. These highly polymorphic ERAPs contain many single nucleotide polymorphisms (SNPs) associated with diseases, including cancer (12). These SNPs can influence aminopeptidase expression, enzymatic activity, and antitumor cytokine expression. Such ERAP mutations may aid tumor cells to avoid immune surveillance and eradication (13).

## The Great Escape

Endoplasmic reticulum chaperones and oxidoreductases can serve as “eat-me” signals on the surface of tumors cells, while promoting tumor growth on others. How ER chaperones escape retention from the ER and move to the plasma membrane remains contentious (14). Several articles within this e-book describe mechanisms to prevent and allow escape of chaperones from the ER and how this influences tumor recognition. Gutiérrez and Simmen describe the regulatory processes involved in retaining or recapturing ER proteins as they attempt to leave the ER (15). Gutiérrez and Simmen describe the conditions by which ER chaperones and oxidoreductases (calreticulin, ERp57, PDI, and GRP94) escape retention and enhance tumor elimination by the immune system. Conversely, other ER proteins (BiP/GRP78) are expressed on many cancer cell surfaces and enhance proliferation, angiogenesis, and therapeutic resistance (16). Undoubtedly, if the “escape” and retention of ER proteins to and from the cell surface can be controlled, the process could be exploited for specific cancer therapies. However, methods to trigger escape of potentially immunogenic regulatory proteins from the ER will have to be strictly regulated, given their ability to modulate tumor growth and induce unwanted adaptive immunity in other diseases. Wiersma and coworkers (5) highlight the fact that in autoimmune diseases, cell stress provokes extracellular release of some ER proteins, which can affect innate and adaptive immune systems and trigger inflammation (17–19).

The idiom “That which hath been is now; and that which is to be hath already been” (*King James Bible, Ecclesiastes 3:15*) is no better illustrated by the fact that parasites have been secreting chaperones for thousands of years as a defense mechanism against the human immune system (20, 21). Ramirez-Toloza et al. (22) describe how surface calreticulin on the Chagas disease causing parasite *Trypanosoma cruzi* blocks activation of complement and aids immune escape of the parasite. Moreover, people with Chagas disease appear less susceptible to certain malignancies (23), and Ramirez-Toloza et al. identify segments of calreticulin that can inhibit tumor angiogenesis.

## War and Peace

Several papers in this e-book describe immune properties of ER proteins capable of raging “war” against tumors. Wang and colleagues describe the adjuvant properties of the stress inducible glucose-regulated protein 170 (GRP170). Previously, they showed an isoform of GRP170 was secreted in melanoma, prostate, and colorectal cancer cells (24–26). GRP170 associates with tumor antigens both intracellularly and extracellularly, acting like a double agent, inducing potent anticancer immunity when outside the cells, but aiding the survival of cancer cells when within the ER. The authors have exploited GRP170 to develop an immune adjuvant for cancer vaccines to trigger a number of adaptive immune processes. An alternative means of delivering antitumor chaperones to the cell surface is by inducing cell stress using photodynamic therapy (PDT) to generate localized production of reactive oxygen species by transfer of light energy from the photosensitizer chlorin C6. This strategy induces surface exposure of calreticulin within minutes of treatment in squamous carcinoma cells (27). Tumoricidal activity is enhanced when PDT treated cells are supplemented with additional recombinant calreticulin. In a similar manner, de Bruyn and coworkers describe that tumor necrosis factor (TNF)-related apoptosis-inducing ligand (TRAIL) recruits CRT to its TRAIL-receptor 2 DISC complex and dissociate CRT from CD47 on the cell surface of cancer cells (28), whereby it may or may not facilitate phagocytic uptake by dendritic cells.

A major aspect of ER protein stimulation of anticancer immunity is to activate specific cytotoxic T cells to provide long lasting immunity against developing tumors. Løset et al. illustrate how tumor-specific T cells armed with specific T-cell receptors (TCRs) could eradicate tumors by interacting with MHC class I containing tumor and/or chaperone peptides (29). Løset and coworkers highlight an alternative therapeutic approach that exploits soluble TCRs that engage peptide/MHC (pMHC) complexes, some of which are now in clinical trials. As an alternative to the stealth-like cancer eradication by TCR-transduced T cells, Graner and colleagues have proposed a more “blanket-bombing” approach. They describe the development of a vaccination rationale comprising of chaperone-rich cell lysates (CRCL) purified from solid tumors designed to induce a plethora of immune responses (30).

## Summary

The ER and its specialized proteins do play a major role in tumor immunity both indirectly and directly. Clearly, there is much more to understand but the potential role and therapeutic options of ER proteins, as described herein, will aid further research into this fascinating topic.

## Author Contributions

Dr. MM, Dr. PE, and Dr. EB have discussed/written the editorial content and approved it.

## Conflict of Interest Statement

The authors declare that the research was conducted in the absence of any commercial or financial relationships that could be construed as a potential conflict of interest.
